# A 27-Year-Old Severely Immunosuppressed Female with Misleading Clinical Features of Disseminated Cutaneous Sporotrichosis

**DOI:** 10.1155/2016/9403690

**Published:** 2016-01-04

**Authors:** Atiyah Patel, Victor Mudenda, Shabir Lakhi, Owen Ngalamika

**Affiliations:** ^1^Department of Medicine, University of Zambia School of Medicine, University Teaching Hospital, Lusaka, Zambia; ^2^Pathology Department, University Teaching Hospital, Lusaka, Zambia; ^3^Dermatovenereology Section, Department of Medicine, University of Zambia School of Medicine, University Teaching Hospital, Lusaka, Zambia

## Abstract

Sporotrichosis is a subacute or chronic granulomatous mycosis caused by fungus of the* Sporothrix schenckii* complex. It is considered to be a rare condition in most parts of the world. It mostly causes cutaneous infection but can also cause multisystemic disease. Unlike most deep cutaneous mycoses which have a primary pulmonary focus, it is usually caused by direct inoculation of the fungus into the skin causing a classical linear, lymphocutaneous nodular eruption. However, atypical presentations of the condition can occur especially in immunosuppressed individuals. We report the case of a severely immunosuppressed female who presented with disseminated cutaneous sporotrichosis which was initially diagnosed and treated as disseminated cutaneous Kaposi's sarcoma.

## 1. Introduction

Sporotrichosis is a subacute or chronic granulomatous mycosis caused by fungus of the* Sporothrix schenckii* complex (including* S. albicans*,* S. brasiliensis*,* S. globosa*,* S. luriei*,* S. mexicana*, and* S. schenckii*) [1]. It occurs worldwide particularly in tropical/subtropical areas and temperate zones with warm and humid climates favoring the growth of saprophytic fungus. Cutaneous infection falls in the category of deep cutaneous mycoses. Unlike most deep cutaneous mycoses, infection is primarily through direct inoculation in the skin rather than dissemination from a primary pulmonary focus.

Since infection occurs following traumatic implantation of the causative fungus (naturally found in soil, plants, hay, and sphagnum moss), the most common clinical presentations include lymphocutaneous and fixed-cutaneous sporotrichosis occurring in persons handling soil or decaying plant material (miners, farmers, gardeners, florists, foresters, etc.) [2, 3]. Occasionally, inhalation of conidia may occur and cause pulmonary and disseminated infection [4]. However, zoonotic transmission of the mycosis from infected animals like cats may also occur.

Disseminated cutaneous sporotrichosis or involvement of multiple visceral organs occurs most commonly in persons with immunosuppression [4]. However, there have been no documented cases of sporotrichosis in Zambia despite having a large burden of HIV disease. In this paper, we report the case of a 27-year-old HIV-positive female with severe immunosuppression who presented with atypical skin lesions of disseminated cutaneous sporotrichosis initially diagnosed and treated as disseminated cutaneous Kaposi's sarcoma (KS).

## 2. Case Report

A 27-year-old female was referred from a primary health care centre to the University Teaching Hospital (UTH) with a 3-week history of ill health. She complained of general body malaise, fever, night sweats, and a skin rash. She described the skin rash as having begun on the nose and subsequently spread to involve the upper limbs and trunk. She was also HIV-positive and had been commenced on antiretroviral therapy at the primary health care centre prior to presentation. Her baseline CD4 count was unknown. She had previously worked as a gardener for several years.

**Figure 1:**
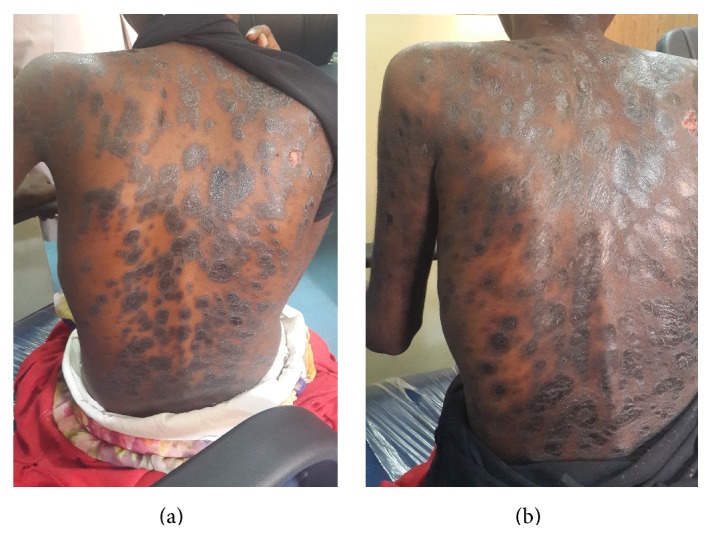
(a) Hyperpigmented plaques (elevated lesions) seen before commencement of treatment; (b) shiny hyperpigmented patches (flat lesions) of postinflammatory hyperpigmentation seen after 3 months of antifungal treatment.

On physical examination, she was pale, chronically ill looking, and wasted. She had multiple, annular, hyperpigmented (purplish-black), slightly raised papules and plaques. A few lesions were ulcerated. The lesions were widespread but mostly involving the face, upper limbs, and trunk (Figure 1(a)). The rest of the examination was unremarkable.

Baseline investigations were done. Chest X-ray was normal. The full blood count revealed severe anemia with a pancytopenia for which she was given a blood transfusion. Upon further questioning, she admitted to receiving a cycle of anticancer chemotherapy. Her renal function tests as well as liver function tests were all normal (Table 1). Her CD4 count was 43 cells/*µ*L. A presumptive clinical diagnosis of disseminated cutaneous KS was made based on the skin lesions and HIV-induced immunosuppression, and a skin biopsy was done. During the course of the admission, the skin lesions were noted to be increasing in number and size. The patient was empirically given triple-agent anticancer chemotherapy for KS whilst awaiting histopathology results. No improvement was noted on anticancer chemotherapy and the patient once again developed severe anemia which was treated with blood transfusion and hematinics.

**Figure 2:**
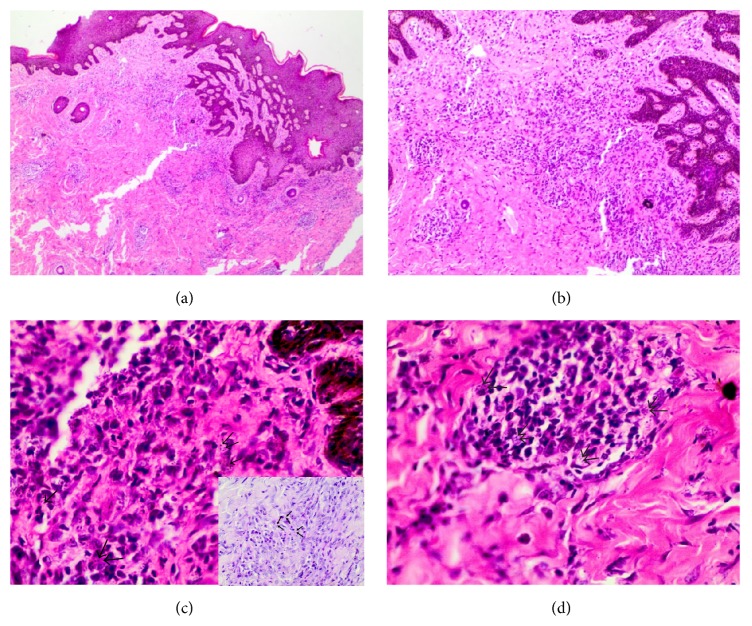
(a) Diffuse inflammatory infiltrate composed of chronic inflammatory cells and macrophages (H&E, ×40); (b) yeast-like forms widely dispersed in the dermis (H&E, ×100); (c) yeast-like forms widely dispersed in the dermis, black arrows pointing out the spores (H&E, ×400). Insert shows a period acid-Schiff stain with arrows pointing out the fungal spores; (d) a granuloma composed of macrophages containing* S. schenckii* organisms (H&E, ×400).

The histology showed a dermal nonspecific mixed inflammatory infiltrate which was predominantly chronic (lymphocytes and plasma cells). In and amongst the aggregates of inflammatory cells were round-shaped yeast organisms consistent with sporotrichosis. The overlying epidermis showed a mild degree of hyperplasia (Figure 2).

It was not possible to do the fungal culture immediately due to unavailability of culture media. The patient had a normal chest X-ray, no central nervous system manifestations, no joint pains, no bony lesions, and no pulmonary symptoms and signs. In the absence of symptoms and signs of other organ systems (despite anemia and pancytopenia attributed to anticancer chemotherapy) the condition was thought to only affect the skin, and no thorough systemic evaluations were indicated.

Our final diagnosis was disseminated cutaneous sporotrichosis. The patient was commenced on Itraconazole 200 mg once daily in addition to her antiretroviral therapy. Improvement in the skin lesions and general condition was noted after three months of therapy. The lesions became flat, and the nodules disappeared, leaving postinflammatory hyperpigmented patches (Figure 1(b)).

## 3. Discussion

Although HIV-infected patients are at increased risk of developing potentially life-threatening disseminated deep fungal infections, sporotrichosis is encountered relatively infrequently [5]. There is limited data on HIV/AIDS and sporotrichosis coinfection. When it does occur, it is mostly disseminated and the CD4 count is usually very low [6].

Our patient presented with clinical and histopathological features highly suggestive of disseminated cutaneous sporotrichosis with no evidence of extracutaneous involvement. Considering that the patient was a gardener, it is possible that she was infected by accidental inoculation of the fungus at the primary site of disease.

Our patient was initially misdiagnosed as a case of KS. This is not surprising considering the purplish-black skin lesions that can easily give an impression of KS and the high prevalence of KS in our setting. Furthermore, the subtype of sporotrichosis that she had, the HIV-induced immunosuppression, and the effect of the anticancer chemotherapy may also have led to the misleading atypical clinical features. In immunocompetent individuals and those with the common classical lymphocutaneous sporotrichosis, clinical diagnosis is usually easy.

Culture is the gold standard in diagnosis and is also the most sensitive [7]. However, when culture is not feasible, histopathology can also be very useful, like in our patient, where characteristic histopathology features can guide the diagnosis [8]. Other fungal organisms that may show a similar histopathological picture include* Histoplasma capsulatum*,* Cryptococcus *species, and* Blastomyces dermatitidis*. Unlike sporotrichosis, histoplasmosis is mainly airborne, and the disseminated form may also affect the mucous membranes. Yeasts of cryptococcosis have variable sizes and appear to have a clear halo on histology, with skin lesions mainly presenting as umbilicated papules with a central hemorrhagic crust.* Blastomyces dermatitidis* infection shows larger, broad-based yeasts on histology, with skin lesions presenting as painless verrucous ulcers. In addition, purely skin involvement without pulmonary involvement is highly unusual in histoplasmosis, cryptococcosis, and blastomycosis.

Treatment for sporotrichosis in immunocompetent hosts is well established. Itraconazole is the drug of choice for cutaneous, lymphocutaneous, and osteoarticular sporotrichosis. Fluconazole can also be used but is less effective than Itraconazole. Amphotericin B is required for severe pulmonary infection and disseminated systemic sporotrichosis [9]. Our patient was commenced on daily Itraconazole with significant clinical improvement noted after about six to eight weeks of treatment. In addition, initiation of highly active antiretroviral therapy was also an integral part in improving clinical response and promoting an adequate immune reconstitution.

Sporotrichosis is regarded to be a very rare disease in Zambia. Nonclassical forms occurring in HIV patients pose a great diagnostic challenge. Clinicians should have a high index of suspicion especially in immunosuppressed individuals who present with atypical skin lesions such as those seen in our patient. When sporotrichosis is suspected, ideally a culture should be obtained. In cases such as ours, where obtaining a culture is not possible, a presumptive diagnosis can be made based on highly suggestive clinical and histopathologic findings and treatment should be initiated. Should the patient not respond to treatment, alternative diagnoses should be strongly considered.

## Figures and Tables

**Table 1:** Initial investigations done at presentation.

Test	Result	Reference range
White cell count	1.56 × 10^9^/L	4.00–10.00
Red cell count	2.73 × 10^12^/L	4.13–5.67
Haemoglobin	6.4 g/dL	12.1–16.3
HCT	24.5%	35.0–47.0
MCV	89.7 fL	79.1–98.9
MCH	23.4 pg	27.0–32.0
MCHC	26.1 g/dL	32.0–36.0
Platelets	88 × 10^9^/L	178–400
Kidney function tests	Normal	—
Liver function tests	Normal	—
CD4 absolute count	43 cells/*μ*L	410–1590
Chest X-ray	Normal	—

HCT: haemotocrit, MCV: mean corpuscular volume, MCH: mean corpuscular haemoglobin, MCHC: mean corpuscular haemoglobin concentration.
